# Identification of DNA repair-related genes predicting pathogenesis and prognosis for liver cancer

**DOI:** 10.1186/s12935-021-01779-1

**Published:** 2021-01-30

**Authors:** Wenjing Zhu, Qiliang Zhang, Min Liu, Meixing Yan, Xiao Chu, Yongchun Li

**Affiliations:** 1 Department of Pharmacy, School of Medicine, Qingdao Municipal Hospital, Qingdao University, Qingdao, 266011 Shandong China; 2 Department of Pulmonary Medicine, School of Medicine, Qingdao Municipal Hospital, Qingdao University, Qingdao, 266011 Shandong China; 3 Department of Orthopedics and Sports Medicine and Joint Surgery, Qingdao Municipal Hospital, Qingdao, Shandong China; 4 Department of Pharmacy, Women and Children’s Hospital, Qingdao, Shandong China

**Keywords:** Liver cancer, mRNA, Biomarker, Prognosis, TCGA

## Abstract

**Background:**

Liver cancer (LC) is one of the most fatal cancers throughout the world. More efficient and sensitive gene signatures that could accurately predict survival in LC patients are vitally needed to promote a better individualized and effective treatment.

**Material/methods:**

422 LC and adjacent normal tissues with both RNA-Seq and clinical data in TCGA were embedded in our study. Gene set enrichment analysis (GSEA) was applied to identify genes and hallmark gene sets that are more valuable for liver cancer therapy. Cox regression analysis was used to identify genes related to overall survival (OS) and build the prediction model. cBioPortal database was used to examine the alterations of the panel mRNA signature. ROC curves and Kaplan–Meier curves were used to validate the prediction model. Besides, the expression of the genes in the model were validated using quantitative real-time PCR in clinical tissue specimens.

**Results:**

The panel of DNA repair-related mRNA signature consisted of seven mRNAs: RFC4 (replication factor C subunit 4), ZWINT (ZW10 interacting kinetochore protein), UPF3B (UPF3B regulator of nonsense mediated mRNA decay), NCBP2 (nuclear cap binding protein subunit 2), ADA (adenosine deaminase), SF3A3 (splicing factor 3a subunit 3) and GTF2H1 (general transcription factor IIH subunit 1). On-line analysis of cBioPortal database found that the expression of the panel mRNA has a wide variation ranging from 7 to 10%. All the mRNAs were significantly upregulated in LC tissues compared to normal tissues (*P* < 0.05). The risk model is closely related to the OS of LC patients. The hazard ratio (HR) is 2.184 [95% CI (confidence interval) 1.523–3.132] and log-rank *P*-value < 0.0001. For clinical specimen validation, we found that all of the genes in the model upregulated in liver cancer tissues versus normal liver tissues, which was consistent with the results predicted.

**Conclusions:**

Our study demonstrated a mRNA signature including seven mRNA for prognosis prediction of LC. This panel gene signature provides a new criterion for accurate diagnosis and therapeutic target of LC.

## Background

Liver cancer (LC) is one of the most fatal cancers throughout the world. It was estimated about 841,080 new cases and 781,631 deaths of liver cancer worldwide in 2020 according to the American Cancer Society [1]. Moreover, rates of both incidence and mortality are 2 to 3 times higher among male than female in most world regions according to the global cancer statistics 2018 in 185 countries [1]. Globally, incidence and mortality of liver cancer in developing countries ranks higher than those in developed countries [2]. The therapeutic effect of liver cancer depends largely on the time interval from diagnosis to treatment, especially for early-stage patients with liver cancer [3]. Some improved treatment methods, such as liver transplantation, hepatectomy and early radiofrequency therapy, have therapeutic value for patients with liver cancer in early stage [4]. However, about more than 70% of patients with liver cancer are diagnosed at advanced stage, which limited the application of conventional therapies [5].

Genomic instability is an important hallmark of cancer, and facilitates the transformation of cancer [6]. Some circulating markers that can predict the response to therapy and the survival of cancer patients have been demonstrated. As reported, miR423-5p can be used as a useful tool to predict response to sorafenib in HCC patients [7]. Moreover, M Caraglia et al. demonstrated that the oxidative stress status and pERK activity in peripheral blood mononuclear cells had high value in the prediction of the response to sorafenib + octreotide therapy in HCC patients [8]. Therefore, understanding the genetic and epigenetic alterations, which are important to hepatocarcinogenesis, is an urgent problem to be solved for providing novel therapeutic targets for HCC [9]. Recent epidemiological studies have reported that two-thirds of cancers are caused by mistakes in DNA replication [10]. Recently, a multi-cohort retrospective analysis revealed that 138 DNA repair genes had prognostic significance in 16 cancer types [11]. DNA repair capacity has a significant correlation with the lymphatic invasion in colorectal cancer patients [12], and DNA Repair promotes drug resistance in ovarian cancer by different hallmark gene sets [13]. Therefore, DNA repair acts as an essential role in maintaining genome stability and cancer development. The deregulation of DNA repair-associated molecules could enhance the resistance of cancer cells to chemotherapy. Genes and proteins related to DNA repair have become therapeutic targets in prostate cancer and ovarian cancer [14, 15]. For the recent study, plenty of gene sets have been reported as a guidance in optimizing the treatment and play an important role as a biomarker in various cancers [16–20]. Multigene prognostic signatures from tumor tissue of patients can predict the prognosis of cancer patients more accurately than a single gene. Especially, multigene prognostic signatures from messenger RNA (mRNA) could provide a better accuracy in cancer prognosis than the non-coding prognostic genes, which enable a better individualized treatment and more effective treatment [21, 22]. And, identifying the mRNAs and hallmark gene sets becomes a prerequisite for clinical application and treatment progress in cancer. However, there is a lack of research on the prognosis of liver cancer with combined markers of mRNAs. Therefore, it is still an urgent problem to find more efficient and sensitive gene sets of mRNAs biomarker for liver cancer.

In this study, Gene set enrichment analysis (GSEA) was used to identify genes that are more valuable for liver cancer therapy and proceeded with further analysis. Finally, 141 DNA repair-related mRNAs were screened out and a seven-gene set that can accurately predict the prognosis of patients with liver cancer was established.

## Methods

### Data sets

The data of patients with LC in TCGA, including mRNA-Seq of transcriptome profiling data and clinical data of the LC patients, was downloaded by the GDC data portal: https://portal.gdc.cancer.gov/. All cases of liver cancer in this study were exclusively hepatocellular carcinoma. The detailed clinicopathological parameters of patients with liver cancer, including age, gender, TNM stage, stage, grade, cancer status and family history of liver cancer, were listed in Table 1.

**Table 1:** Clinical pathological parameters of liver cancer patients in TCGA database

Clinical pathological parameters	N	%
Age		
≤ 61	195	52
> 61	181	48
Tissue		
Adjacent noncancerous tissue	50	12
Liver cancer	377	88
Gender		
Male	255	68
Female	122	32
TNM stage		
T1 + T2	280	75
T3 + T4	94	25
Stage		
Stage1 + stage2	262	74
Stage3 + stage4	91	26
Grade		
Grade1 + grade2	235	63
Grade3 + grade4	137	37
Cancer status		
Tumor free	236	68
With tumor	113	32
Family history of liver cancer		
No	212	65
Yes	114	35
Paired samples from China		
0 HCC nodule	2	40
1 HCC nodule	2	40
2 HCC nodules	1	20

Data sets of GSE101685 and GSE101728 was down loaded from GEO database (https://www.ncbi.nlm.nih.gov/geo/). The website of KM plots online analysis (http://kmplot.com/analysis/index.php?p=service&cancer=liver_rnaseq).

### Patients and clinical specimens

We recruited 5 pairs of matched liver cancer tissues and adjacent normal tissues from Chinese Institution. Among of them, one patient had 2 HCC nodules, and the other patients had 0, 0, 1 and 1 HCC nodule respectively. The tissue samples and corresponding clinical pathology data were from Qingdao Municipal Hospital. This study was approved by Institutional Review Board of Qingdao Municipal Hospital. The number of the approval of this study by the ethical committee is No.109. And the approval document was approved on December 7th, 2019.

### RNA isolation and quantitative real-time PCR (qRT-PCR)

For tissue RNA isolation, 1 mL TRIzol (Invitrogen) was added to 50 mg of tissue and total RNA samples were extracted according to the manufacturer’s instructions. Purified RNA was quantified using NanoDrop 2000 (Thermo Scientific).

cDNAs were synthesized from total RNAs by using ReverTra Ace qPCR RT Kit (Toyobo Co., LTD, Japan). qRT-PCR of GAPDH (glyceraldehyde-3-phosphate dehydrogenase), RFC4, ZWINT, UPF3B, NCBP2, ADA, SF3A3, and GTF2H1 was performed with the SYBR qPCR Mix (Toyobo Co., LTD, Japan). 10 μL reaction system was set up according to the manufacturer’s instructions and amplified for 40 cycles. The expression levels were normalized by GAPDH. Relative expression was calculated using the method of 2^−ΔΔCt^. Primer names and primer sequences are listed in table.

**Table:** 

Primer name	Primer sequence
GAPDH forward	CAGGAGGCATTGCTGATGAT
GAPDH reverse	GAAGGCTGGGGCTCATTT
RFC4 forward	TAAGTCTCCTGGGCCCGTTA
RFC4 reverse	TGCATGGTACTTCACCCAGT
ZWINT forward	GTGGCAGCTACAACAGGAGA
ZWINT reverse	CAGCTTACCCTCTGCAGCTT
UPF3B forward	ATCGAATAAGAAACAAGGATCGTC
UPF3B reverse	AGGCCATCTGGACTTATCACT
NCBP2 forward	CTCTGCACTATGTCGGGTGG
NCBP2 reverse	TGGCGTTTTCCGCATAGCTT
ADA forward	CCAAAGAGGGCGTGGTGTAT
ADA reverse	GTGAGGTCCCCTTCAGCCT
SF3A3 forward	CCATGCAAGATATCTGTGTGCC
SF3A3 reverse	TCTGTGAACTCCACCAAGTTTTG
GTF2H1 forward	GGAACTCGACCGGATCCAAC
GTF2H1 reverse	ATGGTGGCTAGAAGGTGCAAT

### Statistical analysis

GSEA was used to screen out hallmark gene sets with |NES|> 1, NOM *P*-val < 0.05 and FDR q-val < 0.25. Univariate Cox regression analysis was used to screen out prognostic genes with *P* values of < 0.001. Next, a prognostic risk score formula was established according to the results of multivariate Cox regression model. Risk score of each LC patients was calculated by the risk score formula = β1*expression of gene 1 + β2*expression of gene 2 + β3*expression of gene 3 + …. + βn*expre ssion of gene n. Paired and unpaired t test were used to compare gene expression in LC cancer tissues and normal tissues. LC patients were divided into high-risk group and low-risk group using the median risk score as the cutoff. Receiver operating characteristic (ROC) curves and KM plot curves were used to validate the prediction model.

The Log-rank (Mantel–Cox) test was used for survival analysis by GraphPad Prism 7.0. SPSS Statistics 24.0 software calculated HRs and 95% confidence intervals (CI). Paired and unpaired t test were used to assess the difference of genes’ expression by GraphPad Prism 7.0. Differences were considered statistically significant when the *P*-value was < 0.05.

## Results

### Primary screening of hallmark gene sets by GSEA

**Fig. 1:**
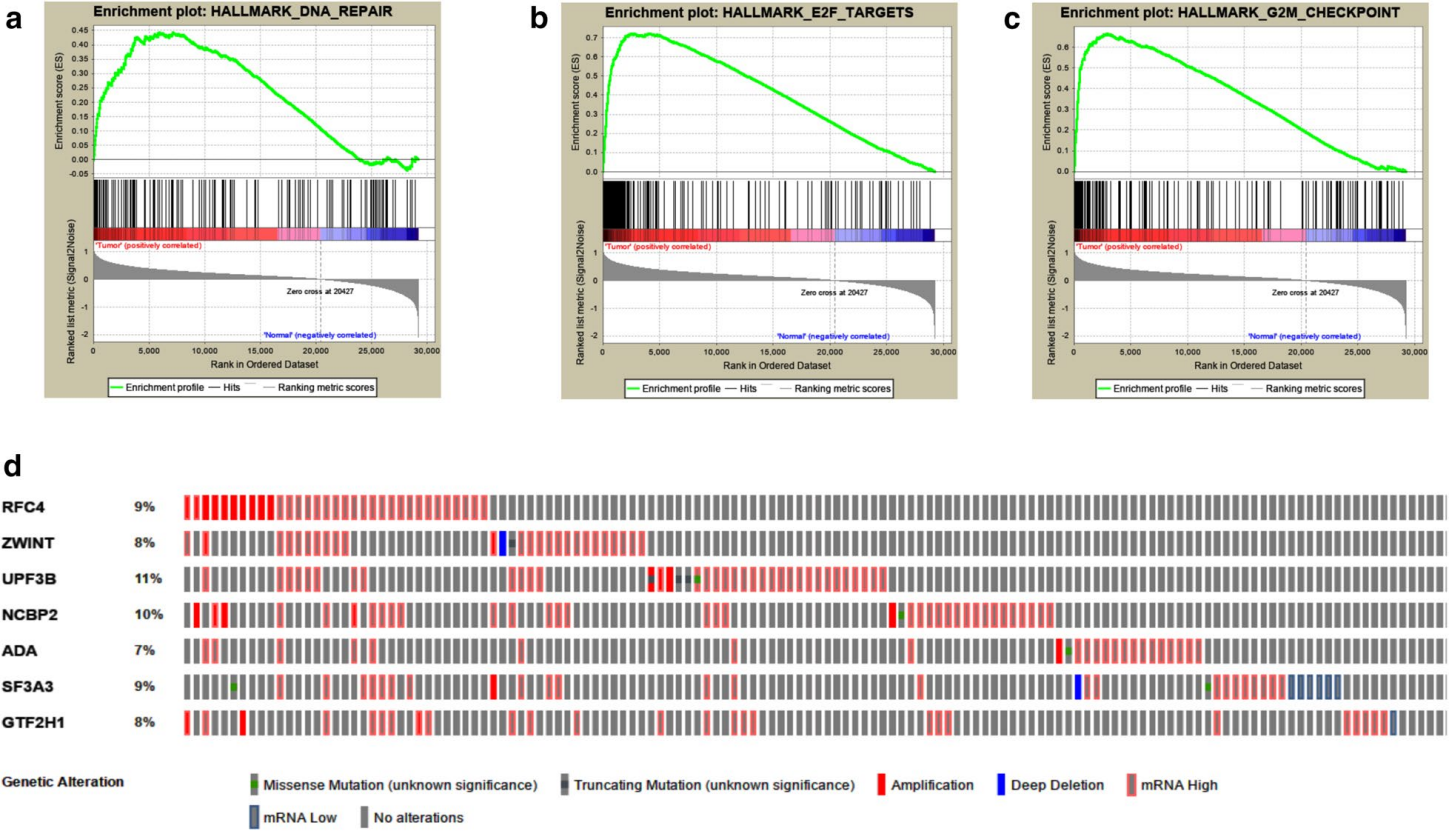
GSEA revealed three gene sets which were significantly differentiated in normal liver tissues versus liver cancer tissues from TCGA.** a** DNA repair gene sets. **b** E2F targets gene sets. **c** G2M checkpoint gene sets. d, the alteration of the selected genes in patients with liver cancer

In TCGA, 422 LC and adjacent normal tissues performed RNA-Seq, and in a total of 29,226 mRNA have expression data. GSEA was used to screen out valuable hallmark gene sets for LC patients. Finally, fourteen abnormal hallmark gene sets in tumor tissue versus adjacent normal tissue were screened out, and three hallmark gene sets, including E2F targets, G2M checkpoint and DNA repair hallmark gene sets, with |NES|> 1, NOM p-val < 0.05 and FDR q-val < 0.25 (Fig. 1a–c and Table 2). Genomic instability is an important hallmark of cancer, and facilitates the transformation of cancer [6]. Recent epidemiological studies have reported that two-thirds of cancers are caused by mistakes in DNA replication [23, 24]. Thus, we select the DNA repair hallmark gene set, which include 141 mRNAs, for further research.

**Table 2:** Hallmark gene sets were enriched in normal liver tissues versus liver cancer tissues from TCGA

GS follow link to MSigDB	SIZE	NES	NOM p-val	FDR q-val	RANK AT MAX
E2F TARGETS	197	2.071552	0.001961	0.007023	4039
G2M CHECKPOINT	195	2.028344	0.005693	0.005554	2901
DNA REPAIR	141	1.649191	0.033333	0.128221	7117

### Identification of prognostic genes related to DNA repair

The results of GSEA showed that a total of 141 genes were obtained in the DNA REPAIR gene sets. We used univariate Cox regression analysis to select prognostic genes with *P* value of < 0.001 from DNA REPAIR hallmark gene sets, and 12 mRNA were screened out. Seven independent LC prognostic indicators, including RFC4, ZWINT, UPF3B, NCBP2, ADA, SF3A3 and GTF2H1, were confirmed by multivariate Cox regression analysis (Table 3). And, some of them have been reported to affect the development of various tumors. ADA encodes an enzyme that catalyzes the hydrolysis of adenosine to inosine in the purine catabolic pathway and elevated in various diseases [25]. RFC4 is involved in DNA replication as a clamp loader, and it’s dysregulation related to the prognosis of patients with liver cancer [26, 27]. Overexpression of ZWINT predicts poor prognosis and promotes the proliferation of hepatocellular carcinoma by regulating cell-cycle-related proteins [28]. SF3A3 encodes subunit 3 of the splicing factor 3a protein complex and act as a novel DNA repair-related prognostic signature in patients with hepatocellular carcinoma [29]. However, in our study, the results of multivariate Cox regression analysis showed that GTF2H1 has the greatest impact on OS of patients with liver cancer. Moreover, it has been reported to strengthen the genetic susceptibility of lung cancer [30].

**Table 3:** The detailed information of multivariate COX survival analysis of the selected prognostic mRNA

gene	Ensemble ID	HR	*B*(COX)	*P* value
RFC4	ENSG00000163918	1.406482	− 0.3093	0.000546
ZWINT	ENSG00000122952	1.359246	0.2752	5.79E−05
UPF3B	ENSG00000125351	1.655998	0.3289	0.000148
NCBP2	ENSG00000114503	2.396939	0.6654	2.02E−05
ADA	ENSG00000196839	1.41584	0.2153	0.000757
SF3A3	ENSG00000183431	2.234263	0.5587	6.27E−05
GTF2H1	ENSG00000110768	2.573995	0.3868	5.18E−05

Further, KM plot online analysis was adopted to analyze the effect of the seven mRNA on the prognosis of patients with liver cancer. The results of KM plot online analysis in accord with Cox regression analysis, LC patients with high expression of these seven genes have more poorly prognosis (*P* value < 0.05, Additional file 1: Figure S1).

The alternation of the selected panel mRNA signature was detected by cBioPortal database. Results showed that the 7 genes screened by us dysregulated in 36% (131 out of 363) of LC patients. UPF3B has the highest rate among all the alternations (Fig. 1d). UPF3B encodes a protein that is part of a post-splicing multiprotein complex involved in both mRNA nuclear export and mRNA surveillance, and it has been reported to have an effect on the prognosis of patients with liver cancer [29].

**Fig. 2:**
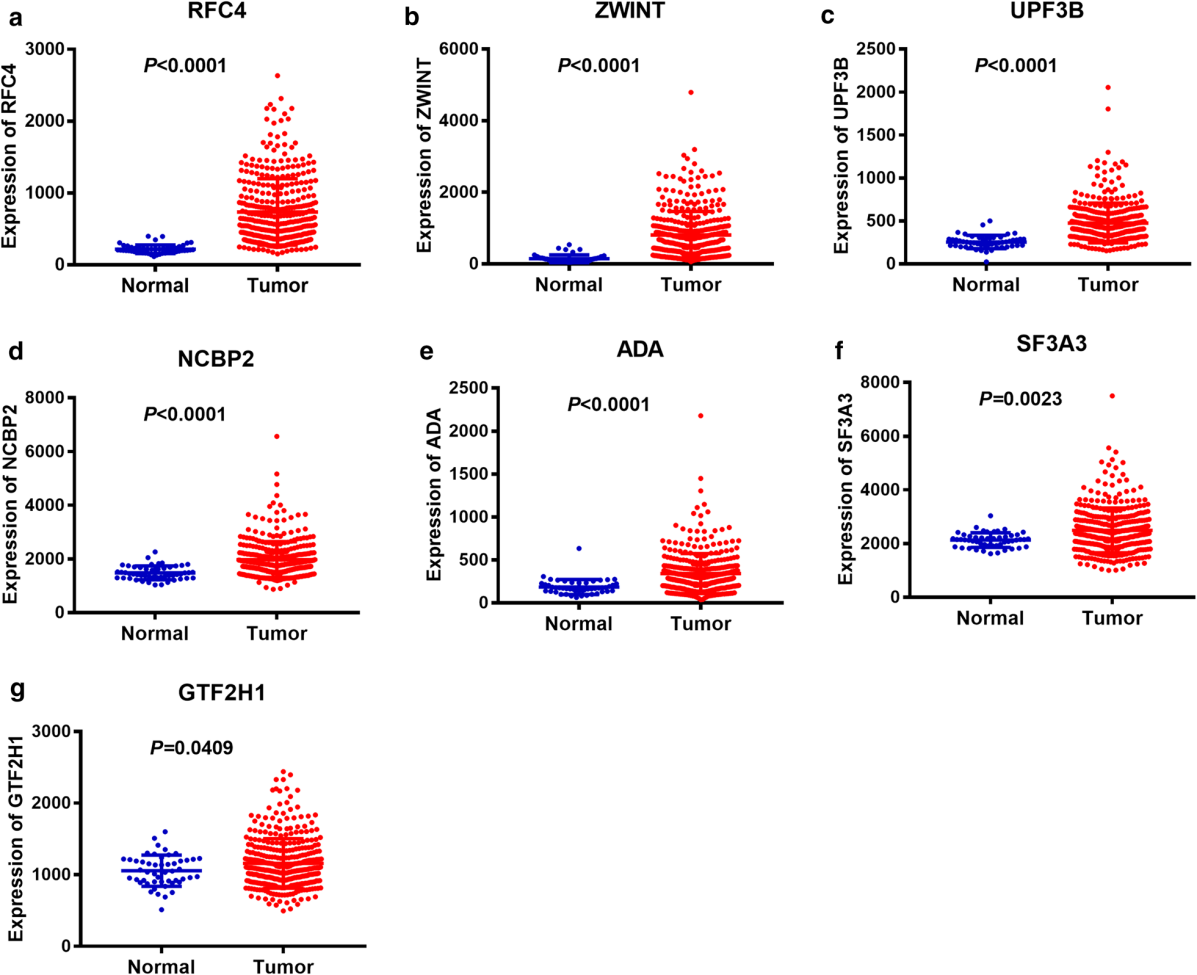
Non-paired t test to detect the different expression of the selected seven genes between LC tissues and normal tissues from TCGA database. **a** RF4. **b** ZWINT. **c** UPF3B. **d** NCBP2. **e** ADA. **f** SF3A3. **g** GTF2H1

**Fig. 3:**
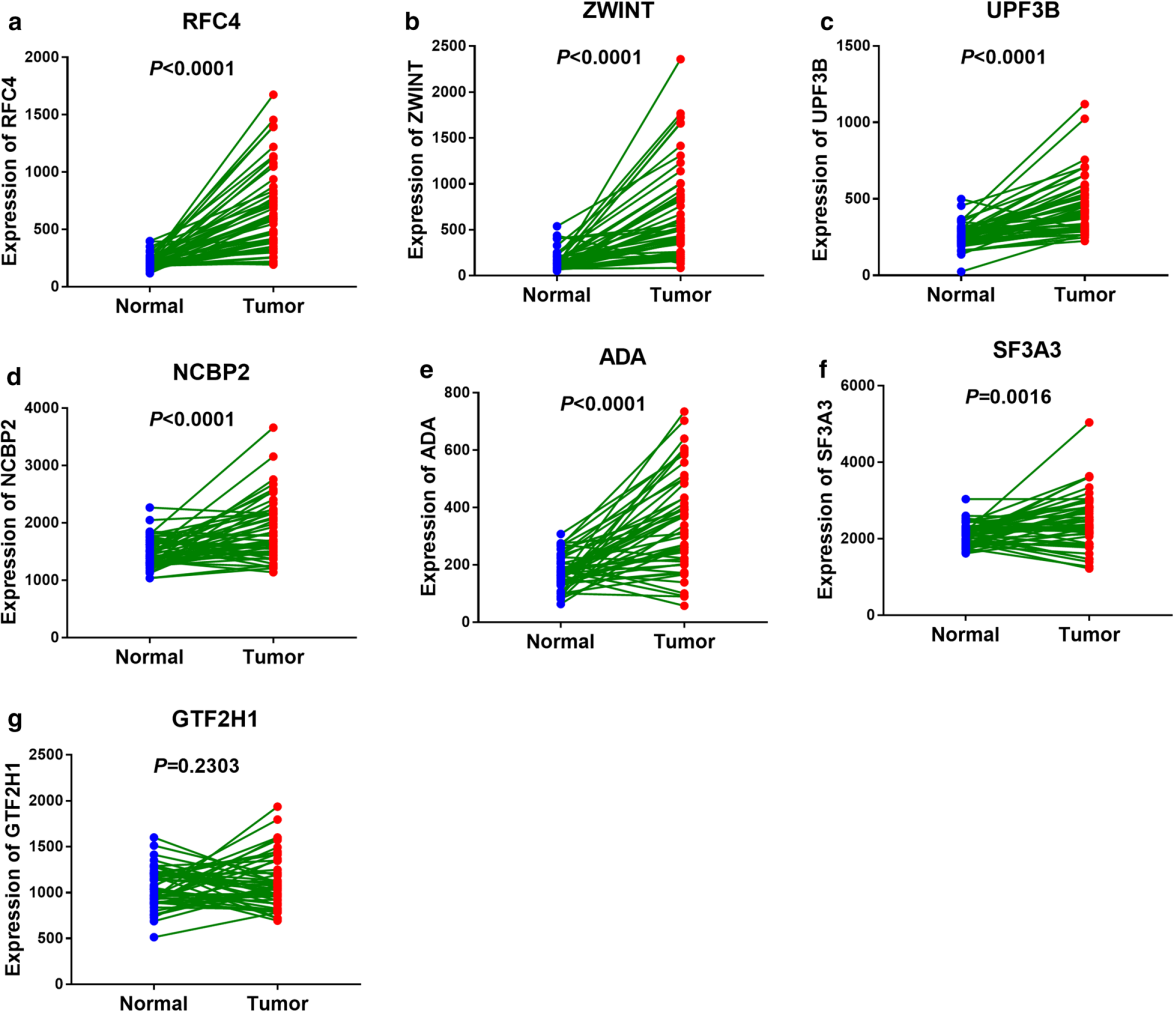
Paired t test to detect the different expression of the selected seven genes in 50 paired samples from TCGA. **a** RFC4. **b** ZWINT. **c** UPF3B. **d** NCBP2. **e** ADA. **f** SF3A3. **g** GTF2H1

Furthermore, we analyzed the differential expression of the seven genes between LC tissues and normal tissues resort to TCGA and GEO database. Unpaired t test showed that all the seven genes we screened have a higher expression in LC tissues versus adjacent normal tissues with a significant differentiation (*P* < 0.05, Fig. 2 and Additional file 2: Figure S2). In addition, we detected the expression of the seven genes in 50 paired samples from TCGA and 7 paired samples from GEO by paired t test. The results of paired t test showed that all the genes’ expression between LC tissues and adjacent normal tissues had statistical differences except GTF2H1 (*P* < 0.05, Fig. 3 and Additional file 3: Figure S3).

### Construction of seven mRNA signature panel for predicting the prognosis of LC patients

**Fig. 4:**
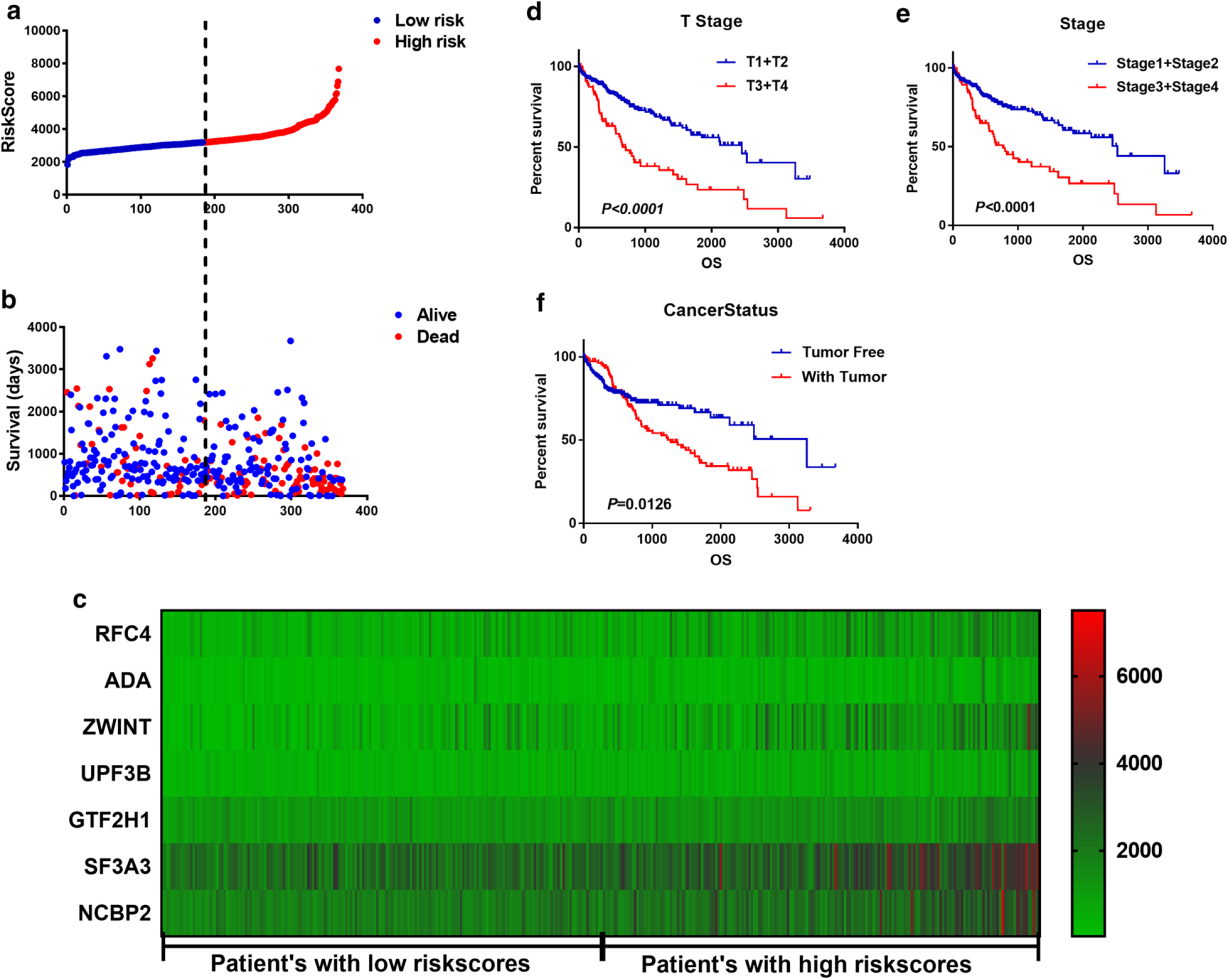
Analysis of clinicopathological parameters affecting prognosis of liver cancer. The data obtained from TGCA databases. **a** the distribution of risk score in patients with liver cancer. **b** the overall survival time and survival status of patients with liver cancer ranked by risk score. **c** the heatmap of the selected seven mRNAs’ expression in patients with liver cancer. **d**–**f** the effect of different clinicopathological parameters including T stage, stage and cancer status on patients’ survival by Kaplan–Meier

The expression level of the selected genes is weighted and then linearly integrated with the regression coefficients obtained by multivariate Cox regression analysis. Risk score = − 0.3093*expression of RFC4 + 0.2153*expression of ADA + 0.3289*expression of UPF3B + 0.2752*expression of ZWINT + 0.3868*expression of GTF2H1 + 0.5587*expression of SF3A3 + 0.6654*expression of NCBP2. The unique risk value for each LC patient was calculated based on the risk score formula. Among of them, NCBP2 has the maximum weighting coefficient and has been reported to interfere with the drug sensitivity of platinum in non-small cell lung cancer [31]. Then, LC patients were divided into low-risk group and high-risk group according to the median value of the risk score (Fig. 4a), and we found that the low-risk group had longer overall survival time and fewer deaths than the high-risk group (Fig. 4b). Moreover, the expression of the selected seven genes in LC patients ascended along with the increase of risk score (Fig. 4c).

### Risk score act as an independent prognostic factor for LC

**Fig. 5:**
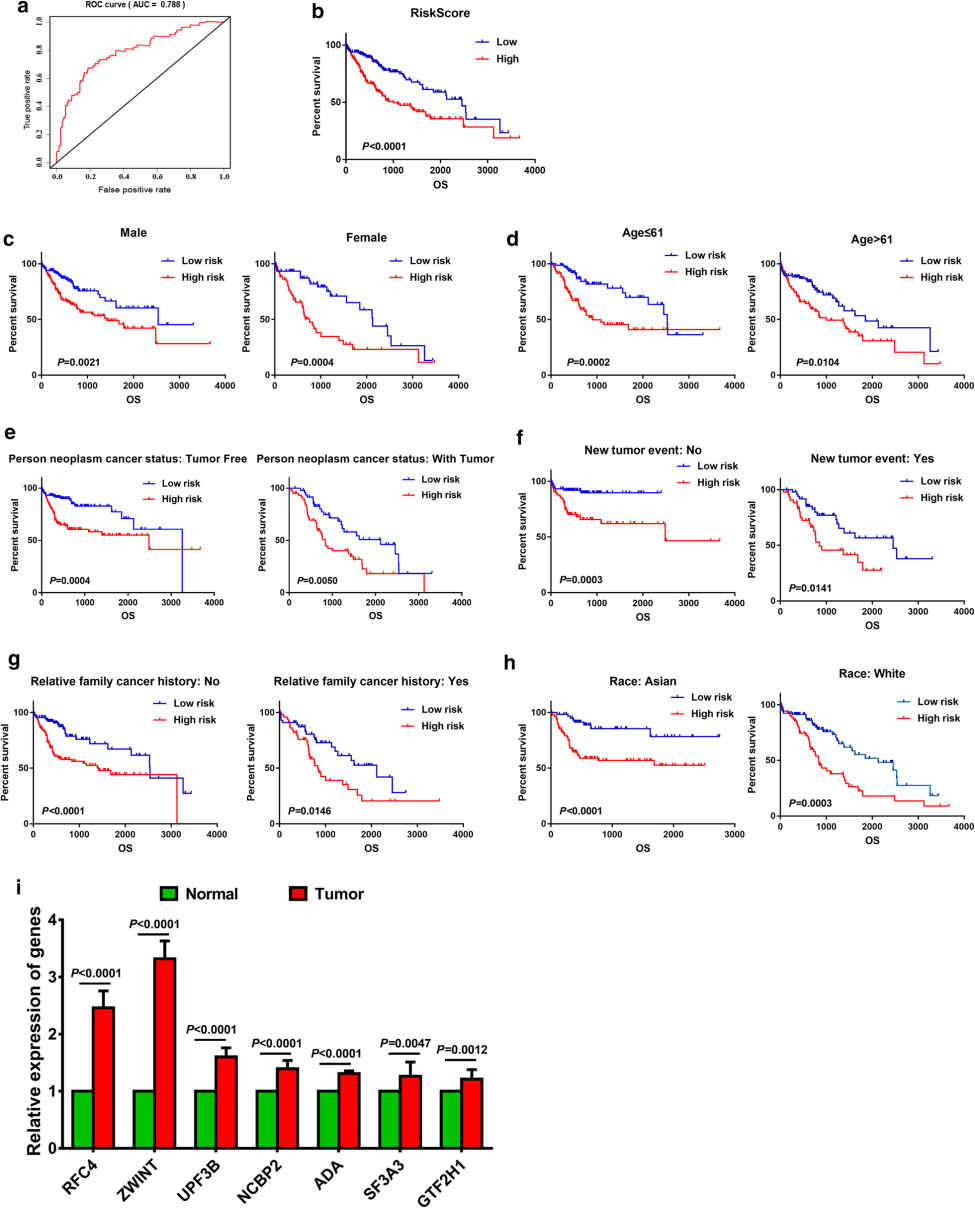
Validation of the mRNA signature panel in patients with LC from TCGA and clinical tissue specimens. **a** ROC curve of patients with liver cancer from TCGA. **b** K–M plot curve for patients with high-risk and low-risk. **c**–**h** stratified analysis of LC patients’ prognosis according to clinicopathological parameters, including sex, age, person neoplasm cancer status, new tumor event, relative family cancer history, race, and risk score. **i** the relative expression of the key genes in clinical liver cancer tissues versus normal liver tissues collected from Chinese Institution

Univariate and multivariate COX regression analysis were performed to confirm the factors affected the prognosis of LC patients. Univariate analysis showed that risk score, new event time, T stage, stage and cancer status were significantly associated with the overall survival time of LC patients. In addition, multivariate COX regression analysis showed that risk score and new event time were independent predictors for the overall survival time of LC patients. Risk score is an important clinicopathological parameter affecting the prognosis of LC patients. It is not only an independent prognostic factor for LC patients, moreover, the mortality rate of patients with high-risk score is 2.184 times as high as that of patients with low-risk score (Table 4). Besides, Kaplan–Meier curves and the log‐rank analysis were then performed to verify the results of COX regression analysis. According to the curves, patients who has a high-risk score, who had later T stage and clinical stage and who are with tumor had poorer prognosis (Figs. 4d–f, 5b). Consequently, the results were self‐consistent, and this observation further proved the accuracy of our analysis.

**Table 4:** Univariate and multivariate Cox analysis to confirm the factors affected the prognosis of patients with liver cancer in TCGA

Factor	Univariate analysis		*P* value	Multivariate analysis		*P* value
	HR	95% CI of HR		HR	95% CI of HR	
Risk score (Low/High)	2.184	1.523–3.132	< 0.0001	2.161	1.302–3.586	0.003
Age	1.012	0.999–1.026	0.076			
Gender (male/female)	0.82	0.575–1.168	0.271			
New event time	0.998	0.997–0.998	< 0.0001	0.998	0.997–0.998	< 0.0001
Tumor topography (T1 + T2/T3 + T4)	2.524	1.774–3.591	< 0.0001	1.251	0.162–9.635	0.83
Stage (stage1 + 2/stage3 + 4)	2.432	1.678–3.525	< 0.0001	0.806	0.105–6.168	0.835
Cancer status (tumor free/with tumor)	1.577	1.099–2.262	0.013	0.611	0.355–1.051	0.075

### Validation of the risk score derived from mRNAs signature

In order to verify the validity of the risk score derived from the seven selected mRNAs, ROC curve and K-M plot curves were adopted. LC patients were divided into two groups according to the median value, then perform a statistical analysis and constructed the ROC curve. The area under the ROC curve is 0.788, and this result indicates that the risk score has high sensitivity and specificity in predicting the prognosis of LC patients (Fig. 5a). Further, we analyzed the survival of the two groups by K-M plot curve, and the prognosis of patients with a low-risk score is much better than that of high-risk score (*P* < 0.0001, Fig. 5b).

Further, a stratified analysis of LC patients’ prognosis was performed to confirm the validity of the risk score. As shown in the K–M plot curves, including clinicopathological parameters of gender (male or female), age (≤ 61 or > 67), person neoplasm cancer status (tumor free or with tumor), new tumor event (no or yes), relative family cancer history (no or yes) and race (Asian or White), patients in high-risk group had poorer prognosis than that in low-risk group (*P* < 0.05, Fig. 5c–h). Besides, the expression of the key genes in the risk model was validated by quantitative real-time PCR in clinical tissue specimens, and we found that all of the genes in the model upregulated in liver cancer tissues versus normal liver tissues, which was consistent with the results predicted (Fig. 5i). These results strongly demonstrate that the risk score derived from the seven mRNAs was a stable and accurate prognostic marker for LC.

## Discussion

Liver cancer (LC) is one of the most common cause of cancer-related death throughout the world [1]. Early diagnosis and novel systemic therapies, including drugs, gene and immune therapies are key factors affecting the prognosis of patients with liver cancer. Previous studies reported that the combination between sorafenib and long acting octreotide is active and well tolerated in patients with advanced hepatocellular carcinoma [32]. Recently, many prognostic non-coding gene expression signatures have been applied to diagnose and predict prognosis of liver cancer [33]. Establishing a specific model by combining multiple genes can identify genes with highly stable diagnostic and prognostic characteristics, and it is a valuable therapeutic target [34]. Moreover, multiple mRNA prognostic sets showed to have a better accuracy in cancer prognosis than non-coding prognostic genes [21, 22]. Consequently, the establishment of prognostic characteristics panel by combining multiple genes is an important step in achieving precise targeted therapy for liver cancer. However, there are few reports on the analysis of prognostic-related mRNA signatures in liver cancer. Hence, we analyzed the expression of mRNA in liver cancer patients and identified valuable hallmark gene sets, including E2F targets, G2M checkpoint and DNA repair hallmark gene sets, by GSEA.

Recent epidemiological studies have shown that two-thirds of cancers are caused by DNA replication errors [10, 23, 24]. Especially, errors in the replication of mRNA, such as mutations in the suppressor gene P53, are particularly important for the development of cancer [35, 36]. Consequently, mutations of DNA repair genes are particularly important in the development of cancer. For example, recent study showed that DNA Repair inhibitor DT01 acts as a novel therapeutic target in colorectal liver metastasis [37]. In our study, we found that DNA repair gene set plays an important role in liver cancer and further analyzed this gene set. In DNA repair gene set, 12 mRNAs are confirmed with *P* < 0.001 by univariate Cox regression analysis. Moreover, among of them, RFC4, ZWINT, UPF3B, NCBP2, ADA, SF3A3 and GTF2H1 are independent prognostic indicators of liver cancer. Unpaired and paired t test showed that all the seven screened genes have a higher expression in LC tissues versus normal tissues with a significant differentiation except GTF2H1 (*P* < 0.05, Figs. 2 and 3).

A marker of multiple genes signature has a more powerful and precise prognostic ability to predict LC patients’ prognosis and maybe an effective classification tool for patients with liver cancer [38, 39]. Therefore, we established a prognostic model for liver cancer using the seven selected genes and divided the patients into two groups by the risk score derived from the prognostic model. We found that the expression of the selected seven genes ascends along with the increase of the risk score and patients in low-risk score group had longer overall survival time and fewer deaths than in high-risk group (Fig. 4b, c).

In addition, the prognostic model of the seven mRNAs signatures was verified by ROC curve and stratified survival analysis. The stratified analysis of different pathological parameters, including sex, age, person neoplasm cancer status, new tumor event, relative family cancer history and race, showed that the risk score derived from the seven mRNA signatures could make a distinction between LC patients with different pathological parameters. Moreover, LC patients in low-risk score group have a longer overall survival time. The results in the stratified analysis are all in accord with integral analysis, which prove the reliability of the risk score derived from the seven mRNA signatures for predicting liver cancer prognosis.

## Conclusions

In summary, the signature of the seven DNA repair-related genes is closely related to the prognosis of patients with liver cancer. And, the model based on the seven genes can be act as an effective tool to predict the prognosis of the LC patients and provide a reference for clinical risk level of the LC patients (Additional files 4, 5, 6).

## Supplementary Information

**Additional file 1: Figure S1.** KM plot curves of the selected seven gene by KM plot online analysis. a, RF4. b, ADA. c, ZWINT. d, UPF3B. e, GTF2H1. f, SF3A3. g, NCBP2.

**Additional file 2: Figure S2.** Non-paired t test to detect the different expression of the selected seven genes between LC tissues and normal tissues from GEO database. a, RF4. b, ZWINT. c, UPF3B. d, NCBP2. e, ADA. f, SF3A3. g, GTF2H1.

**Additional file 3: Figure S3.** Paired t test to detect the different expression of the selected seven genes in 7 paired samples from GEO. a, RF4. b, ZWINT. c, UPF3B. d, NCBP2. e, ADA. f, SF3A3. g, GTF2H1.

**Additional file 4.** The raw data of GSE101685 from GEO database.

**Additional file 5.** The raw data of GSE101728 from GEO database.

**Additional file 6.** The raw data from TCGA database and the raw data of qRT-PCR.

## Data Availability

The datasets analyzed during the current study are available in the TCGA repository, https://cancergenome.nih.gov/.

## Abbreviations

LC: Liver cancer
TCGA: The Cancer Genome Atlas
GSEA: Gene set enrichment analysis
OS: Overall survival
ROC: Receiver operating characteristic
K-M: Kaplan–Meier
HR: Hazard ratio
mRNA: Messenger RNA
RFC4: Replication factor C subunit 4
ZWINT: ZW10 interacting kinetochore protein
UPF3B: UPF3B regulator of nonsense mediated mRNA decay
NCBP2: Nuclear cap binding protein subunit 2
ADA: Adenosine deaminase
SF3A3: Splicing factor 3a subunit 3
GTF2H1: General transcription factor IIH subunit 1
GAPDH: Glyceraldehyde-3-phosphate dehydrogenase
