# Supplementary figures and images for: Identification of DNA repair-related genes predicting pathogenesis and prognosis for liver cancer
Source: Cancer Cell Int. 2021 Jan 30;21:81. doi: 10.1186/s12935-021-01779-1 (PMC7847017; doi:10.1186/s12935-021-01779-1)

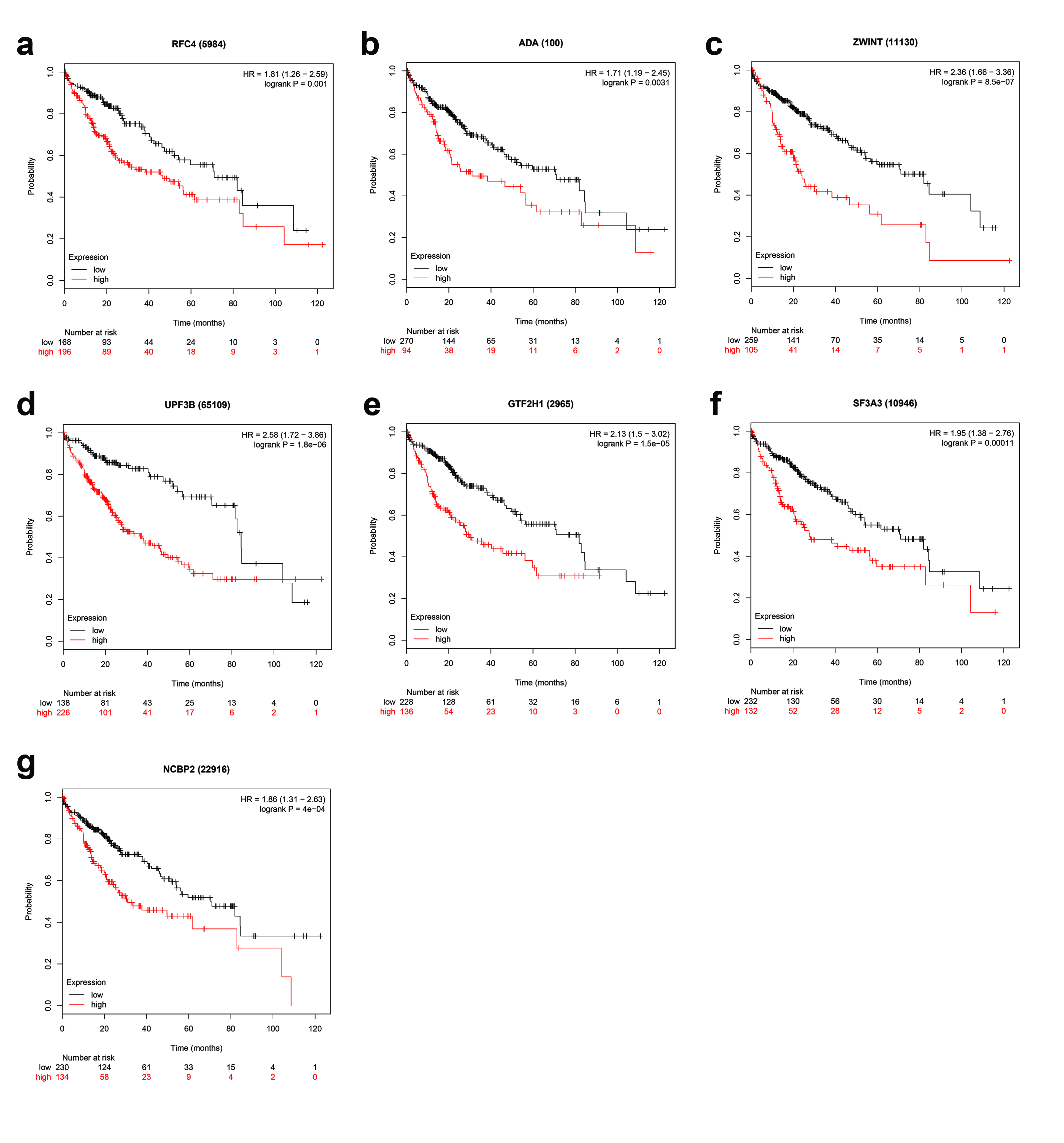

Supplement: Supplementary file 1 — Additional file 1: Figure S1. KM plot curves of the selected seven gene by KM plot online analysis. a, RF4. b, ADA. c, ZWINT. d, UPF3B. e, GTF2H1. f, SF3A3. g, NCBP2. [file 12935_2021_1779_MOESM1_ESM.tif]

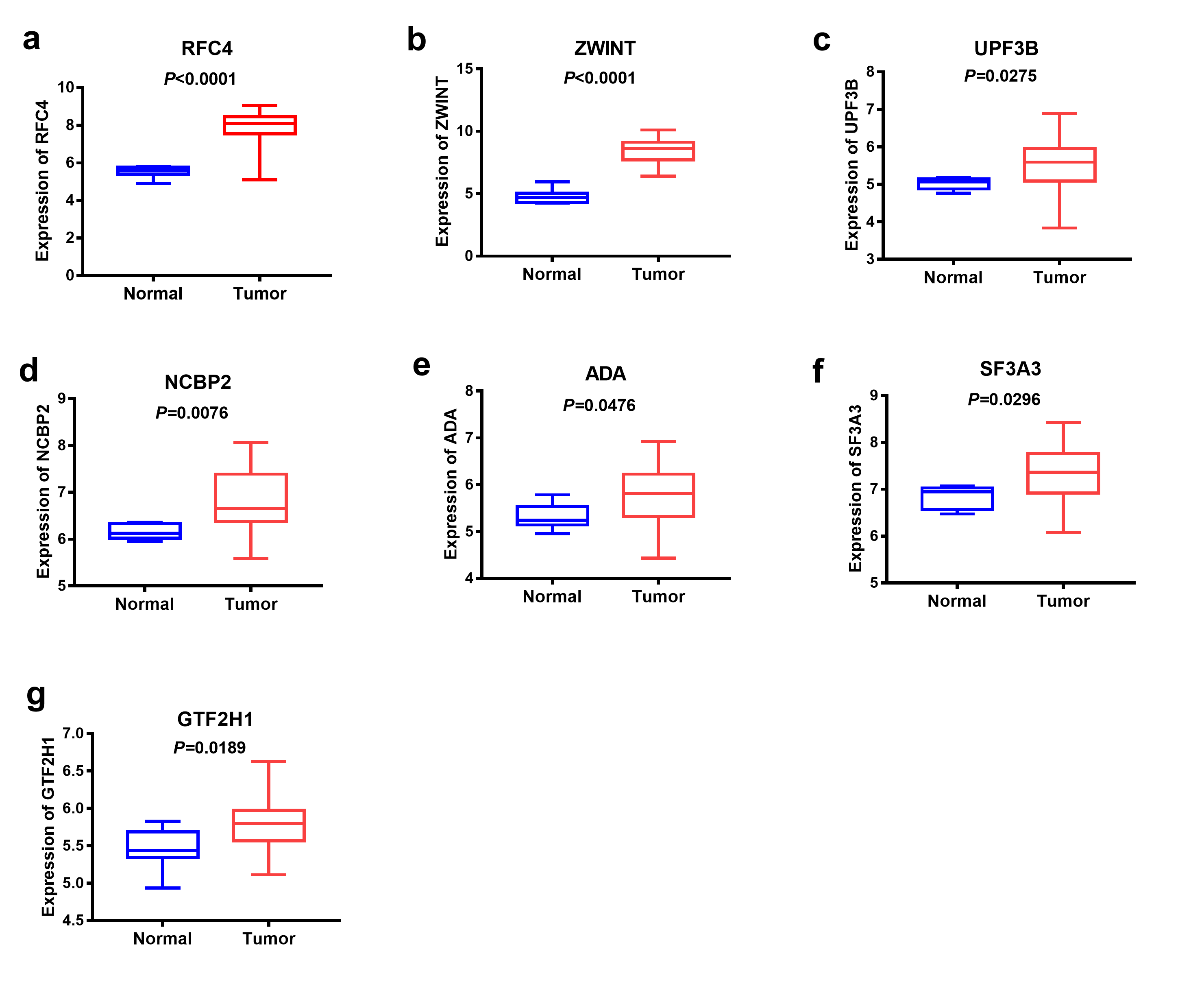

Supplement: Supplementary file 2 — Additional file 2: Figure S2. Non-paired t test to detect the different expression of the selected seven genes between LC tissues and normal tissues from GEO database. a, RF4. b, ZWINT. c, UPF3B. d, NCBP2. e, ADA. f, SF3A3. g, GTF2H1. [file 12935_2021_1779_MOESM2_ESM.tif]

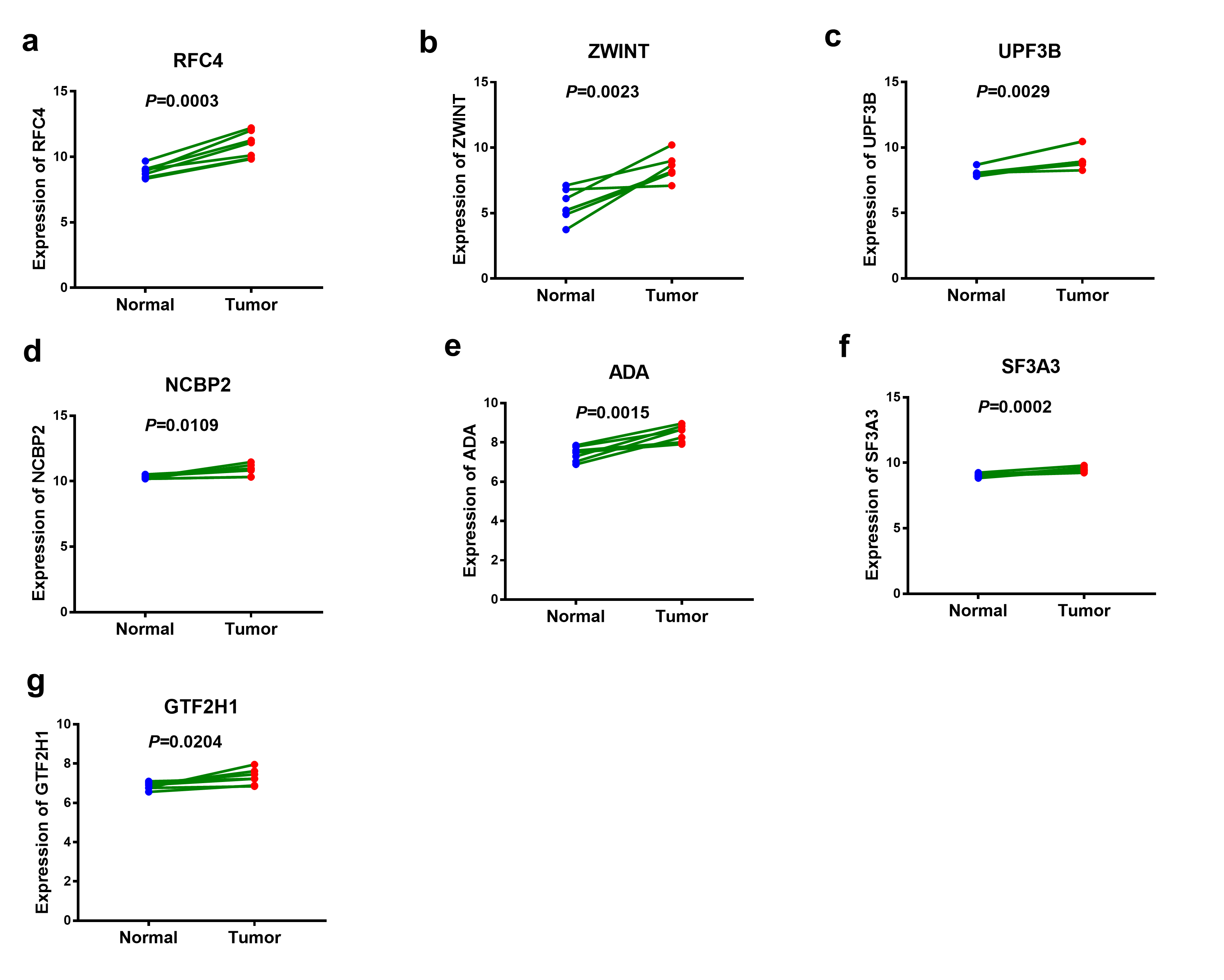

Supplement: Supplementary file 3 — Additional file 3: Figure S3. Paired t test to detect the different expression of the selected seven genes in 7 paired samples from GEO. a, RF4. b, ZWINT. c, UPF3B. d, NCBP2. e, ADA. f, SF3A3. g, GTF2H1. [file 12935_2021_1779_MOESM3_ESM.tif]
